# 

**Published:** 1889-10

**Authors:** 


| | PAGE |
|---|---|
| OSTEOLOGICAL STUDIES OF THE SUBFAMILY ARDEINÆ. R. W. Shufeldt, M.D., C.M.Z.S. | 287 |
| TUBERCULOSIS IN VERTEBRATES. Walter K. Sibely, M.B., Camb | 318 |
| SUCCESSFUL OPERATION FOR THE CURE OF LARYNGISMUS PARALYTICUS OR ROARING IN THE HORSE. J. S. Butler, V.S. | 329 |
| ADDRESS TO UNITED STATES VETERINARY MEDICAL ASSOCIATION. Rush S. Huidekoper, Vet | 332 |
| REPORT OF THE COMMITTEE ON CONTAGIOUS AND INFECTIOUS DISEASES. A. W. Clement, V.S. | 351 |
| GENERAL LYMPHANGITIS J. P. Klench, V.S. | 360 |
| CASE DEPARTMENT | 374 |
| EDITORIAL DEPARTMENT | 382 |
| PROCEEDINGS OF VETERINARY SOCIETIES | 385 |
| REVIEW DEPARTMENT | 396 |
| BOOKS AND PAMPHLETS RECEIVED | 407 |

